# Supplementary material for: Complexome profiling of the Chlamydomonas psb28 mutant reveals TEF5 as an early PSII assembly factor
Source: Plant Cell. 2025 Mar 17;37(6):koaf055. doi: 10.1093/plcell/koaf055 (PMC12164590; doi:10.1093/plcell/koaf055)
Supplement: koaf055_Supplementary_Data [file koaf055_supplementary_data.zip › psb28_interactive.zip!/plots/intensity/qualityPlots/peptides/NFS1_Cre02.g146250.t1.2.html]

### Cre02.g146250.t1.2

Trivial name: NFS1  
SeqCoverage [median]: 30.60  
Mass: 50.20 kDa  
  
 

| Sequence | MissedCleavage | Mass [Da] | wtIntensity | muIntensity | Score |
| --- | --- | --- | --- | --- | --- |
| AIDMTVSHVNK | 0 | 1213.61 | 0.00 | 926.61 | 72.01 |
| ALGVEEELAHTSIR | 0 | 1523.79 | 0.00 | 5820.38 | 72.70 |
| EIIFTSGATESNNTAIK | 0 | 1794.90 | 0.00 | 610.57 | 126.10 |
| ELQADLAHVR | 0 | 1150.61 | 571.65 | 1722.48 | 68.89 |
| GIGALYVR | 0 | 847.49 | 758.15 | 2399.37 | 98.42 |
| HVITTQTEHK | 0 | 1192.62 | 0.00 | 462.42 | 128.81 |
| IPVDVNK | 0 | 783.45 | 0.00 | 1594.23 | 76.03 |
| LEALMSGGGQER | 0 | 1246.60 | 349.03 | 0.00 | 100.92 |
| TGITSQLQGVHLNGPADPAR | 0 | 2031.05 | 0.00 | 1272.22 | 52.78 |
| VIDAMLPYMTEQFGNPHSR | 0 | 2205.03 | 0.00 | 3296.82 | 87.16 |
| VRLEALMSGGGQER | 1 | 1501.77 | 591.87 | 724.38 | 48.11 |
|  |  |  | 2270.7 | 18829.5 |  |
